# Getting “Back on Track” After a Cardiac Event: Protocol for a Randomized Controlled Trial of a Web-Based Self-management Program

**DOI:** 10.2196/34534

**Published:** 2021-12-23

**Authors:** Michelle C Rogerson, Alun C Jackson, Hema S Navaratnam, Michael R Le Grande, Rosemary O Higgins, Joanne Clarke, Barbara M Murphy

**Affiliations:** 1 Australian Centre for Heart Health North Melbourne Australia; 2 Faculty of Health Deakin University Geelong Australia; 3 Centre on Behavioral Health University of Hong Kong Pokfulam China; 4 Department of Psychology Deakin University Geelong Australia; 5 Department of Physiotherapy University of Melbourne Melbourne Australia; 6 Department of Psychology University of Melbourne Melbourne Australia

**Keywords:** coronary heart disease, heart disease, coronary, cardiovascular, prevention, RCT, randomized control trial, secondary prevention, self-management, online, randomised controlled trial, health behaviours, health behaviour, health behavior, depression, cognitive behaviour therapy, motivational interviewing

## Abstract

**Background:**

After a cardiac event, a large majority of patients with cardiac conditions do not achieve recommended behavior change targets for secondary prevention. Mental health issues can also impact the ability to engage in health behavior change. There is a need for innovative, flexible, and theory-driven eHealth programs, which include evidence-based strategies to assist patients with cardiac conditions with their recovery, especially in behavioral and emotional self-management.

**Objective:**

The aim of this study is to determine the short- and longer-term behavioral and emotional well-being outcomes of the *Back on Track* web-based self-management program. In addition, this study will test whether there is enhanced benefit of providing one-on-one telephone support from a trained lifestyle counselor, over and above benefit obtained through completing the web-based program alone.

**Methods:**

People who have experienced a cardiac event in the previous 12 months and have access to the internet will be eligible for this study (N=120). Participants will be randomly assigned to one of the two study conditions: either “self-directed” completion of the *Back on Track* program (without assistance) or “supported” completion of the *Back on Track* program (additional 2 telephone sessions with a lifestyle counselor). All participants will have access to the web-based *Back on Track* program for 2 months. Telephone sessions with the supported arm participants will occur at approximately 2 and 6 weeks post enrollment. Measures will be assessed at baseline, and then 2 and 6 months later. Outcome measures assessed at all 3 timepoints include dietary intake, physical activity and sitting time, smoking status, anxiety and depression, stage of change, and self-efficacy in relation to behavioral and emotional self-management, quality of life, and self-rated health and well-being. A demographic questionnaire will be included at baseline only and program acceptability at 2 months only.

**Results:**

Recruitment began in May 2020 and concluded in August 2021. Data collection for the 6-month follow-up will be completed by February 2022, and data analysis and publication of results will be completed by June 2022. A total of 122 participants were enrolled in this study.

**Conclusions:**

The *Back on Track* trial will enable us to quantify the behavioral and emotional improvements obtained and maintained for patients with cardiac conditions and, in particular, to compare two modes of delivery: (1) fully self-directed delivery and (2) supported by a lifestyle counselor. We anticipate that the web-based *Back on Track* program will assist patients in their recovery and self-management after an acute event, and represents an effective, flexible, and easily accessible adjunct to center-based rehabilitation programs.

**Trial Registration:**

Australian New Zealand Clinical Trials Registry ACTRN12620000102976; http://www.anzctr.org.au/Trial/Registration/TrialReview.aspx?id=378920&isReview=true

**International Registered Report Identifier (IRRID):**

DERR1-10.2196/34534

## Introduction

### Background

Cardiovascular disease is the primary cause of death and disability, both globally [1] and in Australia [2]. With improvements in medical care, increasing numbers of people survive their first cardiac event; however, those who survive are at increased risk of a subsequent event and premature death [3]. The major focus of preventive cardiology is the recovery and rehabilitation of these survivors, with a view to reducing re-events and premature mortality [4].

Secondary prevention, including behavior modification and mood management, is essential for people who have experienced a cardiac event [5]. Up to 90% of the overall risk of acute myocardial infarction (AMI) is attributed to modifiable factors including, physical inactivity, poor diet, cigarette smoking, and medication nonadherence [6]. The survival benefits of increased physical activity, dietary change, and smoking cessation in patients with cardiac conditions have been demonstrated in numerous studies [7,8]. In addition, emotional well-being is essential for the overall health of those with cardiac conditions. Those who experience postevent mental health problems such as anxiety or depression have a poorer recovery than their nondistressed counterparts, with higher rates of hospital readmission [9], more re-events [10], and earlier mortality in the years following [11,12].

Unfortunately, despite the known benefits of behavior change, a large majority of cardiac event survivors do not achieve recommended targets for secondary prevention, with high rates of unhealthy diets, physical inactivity, and resumption of smoking in the postevent year [4,13,14]. Even among those who attend cardiac rehabilitation (CR) programs, initial lifestyle changes are rarely sustained [15]. Mental health problems, such as depressed mood [16] and cognitive barriers, such as negative thoughts [17], also decrease cardiac patients’ ability to undertake health-enhancing behavior change.

While CR is recommended for all cardiac event survivors after an acute event [18] and has been shown to improve health outcomes [19,20], center-based programs are underutilized with evident low participation rates [21,22]. In Australia, research has demonstrated that only approximately 30% of eligible people attend CR programs within 10 weeks of discharge [23]. Nonattendance is partly attributable to access difficulties, with travel time and distance from the venue, and limited car access being key barriers [24]. Regional and rural-based patients with cardiac conditions, as well as those who are younger and have returned to work, are particularly disadvantaged in terms of access to center-based support [25].

Up to 50% of cardiac event survivors have expressed the desire for flexible alternatives to center-based programs, many preferring home-based options, including eHealth [26]. Systematic reviews of telehealth interventions for people with coronary heart disease (CHD), including both telephone- and internet-based interventions, indicate that these programs provide effective risk factor reduction and secondary prevention [27,28]. Research on the effectiveness of web-based support for people with CHD has demonstrated improvements in behavioral and physiological indicators [29-31]. Emerging evidence suggests that outcomes can be enhanced by the addition of concurrent one-on-one telephone support from a health professional [32]. Current estimates suggest that almost 90% of Australian adults are active internet users [33] and that approximately 50% of users, including those of older ages, access health-related information [34], highlighting the potential for the use of web-based health programs with this population.

To our knowledge, there are no programs that aim to equip people with CHD with cognitive and behavioral skills to maintain psychosocial and behavioral health in the long term. People need evidence-based strategies and tools to develop skills to self-manage their health in the longer term [15]. Research has consistently highlighted the importance of theory-driven programs in assisting people with behavior change [35]. For example, self-regulation theory, defined as a goal guidance process aimed at the attainment and maintenance of personal goals [36], underpins several behavioral interventions to support change, such as self-monitoring, feedback, reward, and goal-setting [37,38]. Likewise, cognitive-behavioral therapy (CBT) has been used successfully to assist people with CHD in self-management of both behavioral [39] and psychosocial [40] risk factors. Motivational interviewing (MI) has also been shown to improve engagement and to decrease resistance [41], and it can enhance CR outcomes [42]. For cardiac patients, self-efficacy of behavior change has been shown to be associated with short- and longer-term maintenance of exercise and smoking cessation [43,44]. Evidence suggests that outcomes are optimal when these theories, interventions, and strategies are integrated [45,46].

With the aim of addressing gaps in secondary preventive care for people with CHD, the Australian Centre for Heart Health (ACHH) developed a face-to-face, group-based self-management program, *Beating Heart Problems,* which was underpinned by a framework of self-regulation theory and patient-centered care. Based on the principles and strategies of CBT and MI, the program was designed to provide cardiac event survivors with the cognitive and behavioral skills to self-manage their mood and health behaviors in the long term. A randomized controlled trial (RCT) of the *Beating Heart Problems* program, involving 275 cardiac event survivors, demonstrated that the intervention group showed a superior reduction in 2-year risk of a recurrent event, greater improvements in functional capacity and behavioral health, and greater reductions in depression incidence and severity, compared to the control group [47,48]. To overcome issues of access, accessibility, and inequities, the face-to-face program was translated for web-based delivery. The web-based program, titled *Back on Track*, is underpinned by the same frameworks and theories as the face-to-face program and includes modules that focus on both behavioral and emotional aspects of recovery. In a pilot study, the web-based *Back on Track* program was shown to be acceptable and useful in terms of self-management support following a cardiac event [49].

### Objectives

The aim of this study is to assess the benefits, in terms of behavioral and emotional well-being, of the web-based *Back on Track* self-management program, and to test whether participants obtain enhanced benefit through the provision of one-on-one telephone support from a trained lifestyle counselor (supported arm), over and above benefit obtained through completing the web-based program alone (self-directed arm). By including 2 follow-up assessments, one on postprogram completion (2 months) and one at 4 months post program (6 months postenrollment), we aim to assess both immediate and sustained benefits.

We hypothesize that participants randomly allocated to each arm of the trial will show significant improvement in their behavioral and emotional well-being, self-efficacy, and self-rated health from the preprogram to postprogram assessments, with benefits sustained 4 months after program completion. We also hypothesize that participants allocated to the supported program will demonstrate superior improvements in behavioral and emotional well-being, self-efficacy, and self-rated health compared to those allocated to the self-directed arm, at both the postprogram and follow-up assessments.

Ethical approval was granted by the Deakin University Human Research Ethics Committee (2019-438). This project has been funded by the HCF Research Foundation. This study will be conducted and reported in accordance with the CONSORT-EHEALTH guidelines [50].

## Methods

### Study Design

This project uses a 2-armed RCT design, with participants who register to be involved in the trial being randomly allocated to one of two arms: either “self-directed” completion of the *Back on Track* program or “supported” completion of the *Back on Track* program. The self-directed arm involves participants completing the web-based *Back on Track* program at their own pace without assistance. The supported arm involves the addition of 2 telephone sessions with a trained lifestyle counselor. The telephone sessions are protocol driven, with a written manual developed by a health psychologist.

**Figure 1:**
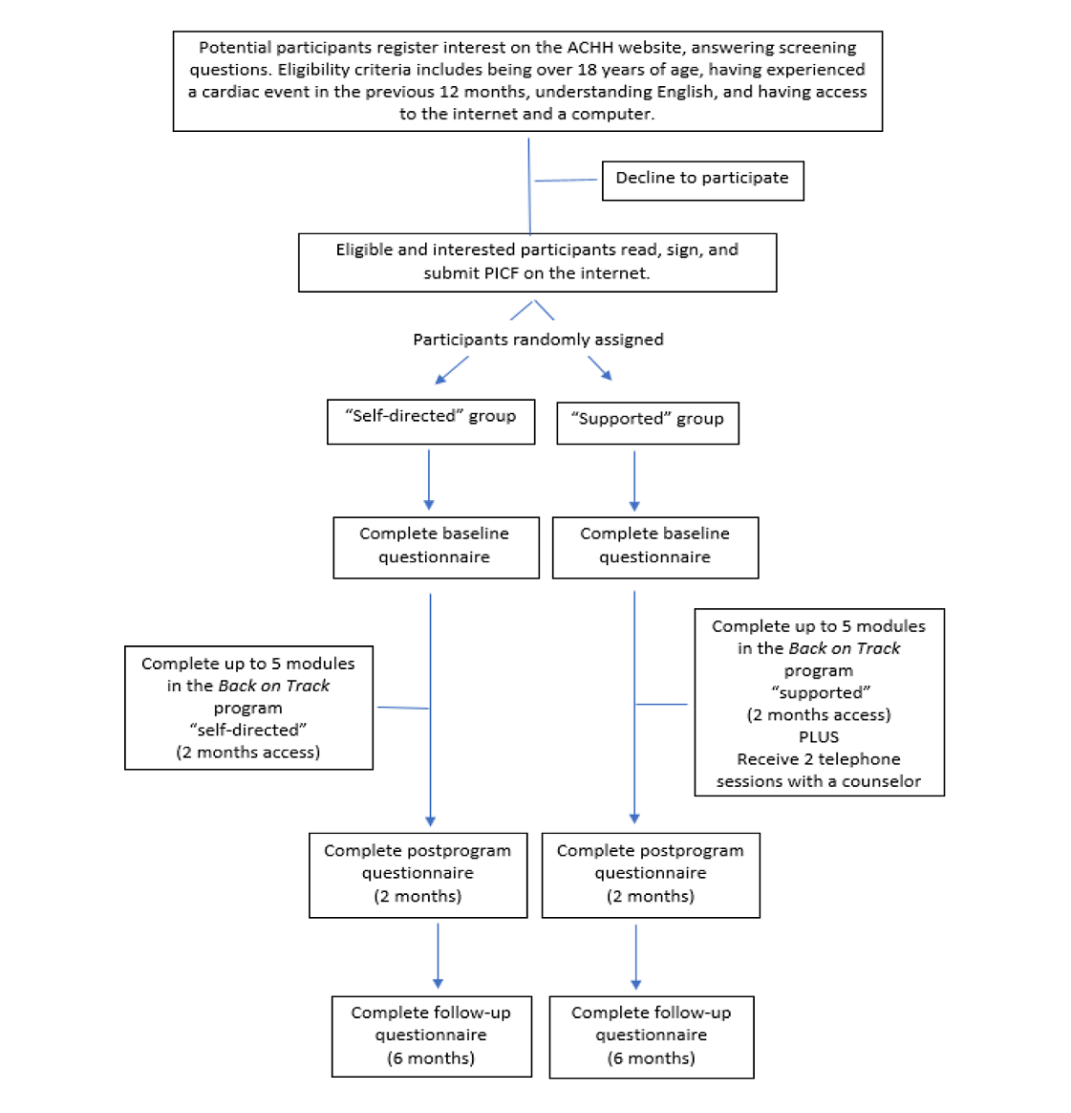
Trial design based on CONSORT (Consolidated Standards of Reporting Trials) requirements. ACHH: Australian Centre for Heart Health; PICF: participant information consent form.

Measures are assessed at three timepoints across the study: a baseline questionnaire is completed prior to participants receiving access to the web-based program, a postprogram questionnaire at the conclusion of the web-based program access (2 months after baseline), and a follow-up questionnaire 4 months later (6 months after baseline). Figure 1 shows a flowchart of the study design and the participant numbers.

An RCT design was chosen for this trial to accurately compare outcomes for the 2 modes of delivery of the web-based *Back on Track* program, either self-directed or supported. By comparing outcomes for participants in the 2 groups, we will be able to determine whether the supported approach provides benefits over and above the self-directed use of the web-based *Back on Track* program. The result will then inform the optimal form of delivery of the *Back on Track* program for future rollout to people with CHD across Australia.

### Recruitment and Participants

A total of 120 participants will be recruited into the trial, with 60 randomly allocated to each of the 2 arms of the trial. Participants will be adults (over 18 years of age) who have had an acute cardiac event, such as AMI, coronary artery bypass graft surgery (CABGS), or percutaneous coronary intervention (PCI), in the past 12 months. Participants are required to be fluent in English to comprehend the *Back on Track* program, which is currently available in English only. Participants are also required to have access to the internet and a computer or tablet.

Participants are being recruited using both direct engagement via a nationwide social media recruitment drive, and indirect engagement via cardiac health professionals and community-based cardiac support organizations, namely HeartBeat Victoria and Heart Support Australia. A promotional flyer is used for all promotion and recruitment. The nationwide social media recruitment drive involves announcements about the trial on internet-based social media platforms such as Facebook, Twitter, and LinkedIn. HeartBeat Victoria and Heart Support Australia also promote the study to their members, who are all people living with heart disease. Details of eligibility criteria are provided on the relevant websites and organization promotional material. Likewise, the trial is being promoted among cardiac health professionals, based on a list of health professionals who are involved in cardiac rehabilitation services across Australia or have attended past ACCH training programs.

In all cases of recruitment, both direct and indirect, potential participants are directed to the ACHH website for detailed information about the study and for instructions on how to register on the internet. Screening for eligibility is undertaken at the time of participant registration via the ACHH website using a set of brief screening questions; specifically, participants are asked to provide their name and contact details, type, and date of cardiac event and to confirm that they are able to read and understand English and that they have access to the internet and a computer or tablet device. All participants are asked to read, sign, and return the participant information and consent form (PICF) after registration and prior to randomization and being given program access. The PICF is emailed to participants via the secure web-based data management and survey program, Research Electronic Data Capture (REDCap), which they sign and return electronically to ACHH.

Once registered, participants are randomly allocated to either the self-directed or the supported arm of the trial using an independent and automatic random allocation numbering system. The person undertaking the random allocation is blind to group and participant details. All participants are then asked to complete the web-based baseline questionnaire, also disseminated using REDCap. The link to questionnaires is emailed to participants at each timepoint. Access to the *Back on Track* program is granted to participants for a 2-month period after they complete the baseline questionnaire.

Participants are asked to recomplete the main outcome measures again post program (2 months after initial access to the program) and at 4-month follow-up (6 months after program access). Again, the REDCap survey link is emailed to participants.

### Sample Size and Power

Our sample size is based on a systematic review of telehealth-based cardiac rehabilitation [28] on lifestyle behaviors, health outcomes, and quality of life, where the effect sizes on promoting healthy lifestyle ranged from 0.42 to 1.29. Taking a conservative approach, we expect a medium effect size of 0.55. Allowing for 80% power and a significance threshold of .05, the estimated sample size is 120 (60 per arm), which takes into consideration an expected attrition rate of 12% based on prior studies with similar posttest time points of 2-4 months [31,51].

### Intervention

#### Back on Track Program

The *Back on Track* program begins with a goal-setting module, followed by 4 self-selected modules relating to (1) healthy eating, (2) physical activity and reduced sitting, (3) smoking cessation, and (4) cardiac blues and depression. Participants in both the self-directed and supported arms are asked to complete modules relevant to them, in their own time and in any order over the course of 2 months. Within each module, participants undertake exercises that enable them to review situations in their lives, and using concepts from CBT, to identify, challenge, and change the unhelpful thoughts and beliefs associated with risk factors and negative emotions. Participants are prompted to develop action plans and coping plans for implementing practical health behaviors, including goal-setting, and identifying motivators for change, resources, barriers, rewards, and relapse prevention strategies. Other activities include using strategies to increase self-efficacy, including stories of role models, and exploring ratings of importance of and confidence to undertake behavior change.

**Figure 2:**
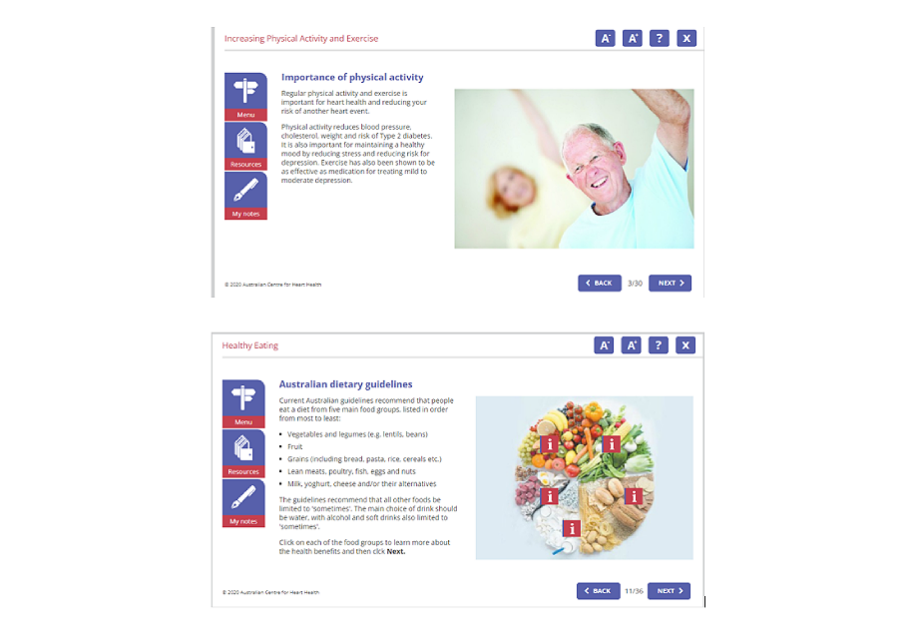
Examples of informative slides in the *Back on Track* program.

**Figure 3:**
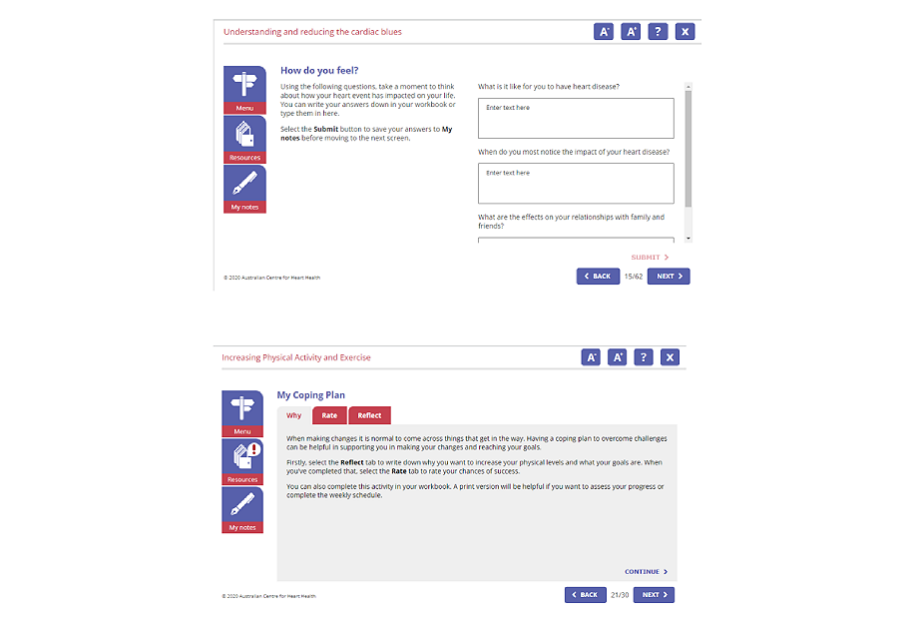
Examples of interactive slides in the *Back on Track* program.

The behavioral recommendations included in the modules (eg, healthy eating, physical activity, and smoking cessation) are all based on the current National Heart Foundation of Australia (NHFA) guidelines [52]. Although not being the central focus of the *Back on Track* program, it is important to present, up-to-date, relevant information on these guidelines and recommendations. This can help participants with aspects of their self-management, including goal-setting. Figures 2 and 3 show screenshots of various aspects of the web-based *Back on Track* modules.

#### Additional Telephone Sessions for the Supported Group

Supported group participants are offered 2 telephone sessions with a trained lifestyle counselor to assist them in goal-setting and overall self-management while undertaking program modules. The session duration is 45-60 minutes for session 1 and up to 30 minutes for session 2. The first session occurs around 1-2 weeks postenrollment. The counselor begins the session with rapport development, then screens for anxiety and depression using the Patient Health Questionnaire-4 (PHQ-4) [53]. Self-efficacy for managing changes associated with health behaviors and emotional well-being is also assessed. Decisional balance and ambivalence are explored.

Motivational interviewing strategies are used to support participants to articulate clear and achievable goals and to identify areas they wish to address in behavioral and emotional self-management. Action and coping planning interventions are used to support translation of goals into actions. At completion of the first session, the second telephone session is scheduled for a mutually agreed time (approximately 4 weeks after the first session). The second telephone session includes a reflection on anxiety or depression symptoms, self-efficacy, coping and action plans, relapse prevention, and maintenance of behaviors. In addition, if the counselor considers that the individual requires more intensive mental health support, referral options for counseling are discussed.

An SMS reminder is sent 24 hours prior to each telephone session appointment to facilitate engagement. To ensure consistency and quality of program delivery, lifestyle counselors receive fortnightly supervision with an experienced registered health psychologist.

### Measures

Sociodemographic information is collected at baseline only. All participants complete the outcome measures of dietary intake, physical activity and sitting time, smoking status, anxiety and depression, stage of change, and self-efficacy in relation to behavioral and emotional self-management, quality of life, self-rated health, and well-being at baseline, post program (2 months after initial access to the program), and at 4-month follow-up (6 months after program access). Program acceptability is assessed post program only using a 6-item scale developed specifically for this study.

#### Primary Outcome Measures

##### Depression

Depression is assessed using the PHQ-9 [54]. The PHQ-9 is a brief 9-item depression screening tool based on the symptoms that comprise a diagnosis of major depressive disorder. Scores range from 0-27, with scores between 0-4 indicating minimal depression, 5-9, mild depression; 10-14, moderate depression; 15-19, moderately severe; and ≥20, severe depression. In patients with cardiac conditions, research has demonstrated an optimal PHQ-9 threshold of ≥6 [55,56], which combined improved sensitivity and retained specificity (sensitivity=83% and specificity=76% [56]) as compared to a threshold of ≥10, for detecting major depressive disorder based on a structured clinical interview. The PHQ-9 has been endorsed by the NHFA as the recommended tool for depression screening [57].

##### Physical Activity and Sitting Time

Physical activity and sitting time are measured using the Active Australia Survey (AAS) [58] with sitting questions from the International Physical Activity Questionnaire (IPAQ) (long form) [59]. The 8-item AAS measures frequency of walking, moderate activity, and vigorous activity in the last 2 weeks. The AAS has acceptable reliability and validity in the Australian community [60] and CR populations [61]. Participants are classified, according to the NHFA, as achieving the recommended target for adequate physical activity for secondary prevention [52] if they have been engaged in ≥150 minutes of physical activity per week combined across the 3 domains assessed. Sitting time is measured using the relevant questions from the IPAQ long form which asks participants about their total time spent sitting on a typical weekday and weekend day [59].

##### Dietary Intake

Diet quality is assessed using the Diet Quality Tool (DQT) [62], a 13-item questionnaire where all questions relate to nutrients of concern in the prevention of CVD (eg, fruit and vegetable, saturated fat, total fat, ω-3 fatty acids, fiber, and salt intake). Each item is scored from 0 to 10, where 10 indicates that the participant is meeting the NHFA’s secondary prevention nutrition guidelines [52]. Participants are classified as achieving a healthy diet for CHD secondary prevention if they receive a total DQT score >60%. This cut-off was originally identified by a panel of four accredited practicing dieticians with clinical, CR, and dietary research methodology experience, and validated against a 4-day food diary in Australian patients with cardiac conditions attending CR [62]. The DQT was found to be a valid and useful dietary assessment tool in a secondary CVD prevention setting [62].

#### Secondary Outcome Measures

##### Smoking Status

Smoking is assessed on the basis of self-reports by asking participants “Do you smoke?” Current smokers are asked how many cigarettes they smoke per day. Former smokers are asked when they quit smoking.

##### Anxiety

Anxiety is assessed using the 7-item Generalized Anxiety Disorder instrument (GAD-7) [63]. The GAD-7 was developed to provide a brief self-report measure to identify generalized anxiety in primary care, and asks participants, using 7-items, to indicate how often they have been bothered by certain problems over the past 2 weeks. The GAD-7 has good reliability and validity for detecting generalized anxiety [64]. The GAD-7 has been validated within a large sample of people in a primary care setting [63] and in cardiac populations [65].

##### Well-being

Well-being is assessed using the World Health Organization–Five Well-Being Index (WHO-5) [66]. This is a short, self-reported measure of current subjective mental well-being. The WHO-5 has adequate validity as an outcome measure in clinical trials and has been applied successfully as a generic scale for well-being across a wide range of study fields [67]. It has also been successfully used as a predictive tool for patients with cardiac conditions [68].

##### Quality of Life and Self-rated Health

Quality of life is measured using the Short Form Health Survey (SF-12) [69]. The SF-12 is a 12-item measure often used to compare health status between 2 groups of people, to identify predictors of health status and to determine health status in a specific disease population [70]. The SF-12 also includes one item that assesses self-rated health, which has been shown to predict survival [71]. The instrument is regarded as a reliable and valid generic measure of health-related quality of life in cardiac populations [72,73].

##### Self-efficacy

Self-efficacy is assessed using an 8-item scale, which has been designed specifically for this study. Participants indicate on a 5-point Likert scale how confident they are that they can make or sustain relevant behavioral and emotional changes.

##### Readiness to Change (Stages of Change)

Participants are asked to indicate their readiness to change relevant behaviors on a 5-point Likert scale from not thinking about making changes to have maintained changes for more than 6 months. This scale has been developed specifically for this study using wording from the Stages of Change model.

##### Additional Measures

Basic sociodemographic (namely age, sex, country of birth, marital status, living arrangement, employment status, educational level, and financial strain), medical (other physical and mental health conditions), and event-related information (event type, date of event, and attendance at cardiac rehabilitation) is collected via a self-report questionnaire, using standard questions used in previous ACHH studies [74].

### Data Analysis

In evaluating outcomes, there will be two groups of participants based on the mode of delivery: “self-directed” and “supported.” All statistical analyses will be performed on an intention-to-treat (ITT) basis with *P*<.05. In the case that ITT produces a null finding, it is possible that this is due to participants in the supported group not participating in the additional telephone sessions (that is, not receiving the intended treatment). To test that hypothesis, we will then undertake treatment-received analysis (also called Per Protocol) whereby only those who received the treatment in accordance with the protocol are included. This will enable us to determine whether any differences were obtained between those who received the self-directed treatment and those who received the supported treatment. *P* values less than .05 will be considered significant for the primary outcome and those less than .01 will be considered significant for secondary outcomes. The effect of the intervention on the primary and secondary outcomes at 6 months will be assessed using analysis of covariance (ANCOVA) models that include the baseline value of the outcome as a covariate and the group assignment (self-directed versus supported) as a categorical variable. The treatment effect, its effect size (Hedge g), and 95% CIs for the treatment effect and within-group changes from baseline to 6 months will be calculated from the ANCOVA models. The sensitivity of the results to missing data will be evaluated using a data-based multiple imputation procedure.

### Primary Outcome Measures: Depression, Physical Activity, and Dietary Intake

As the main outcomes, we will examine differences between the 2 groups in the proportion of participants who are:

1. classified as *nondepressed* at baseline, post program, and follow-up (based on a PHQ-9 score of <6) [55,56],
2. achieving physical activity guidelines of ≥150 minutes of physical activity per week [52] at baseline, post program, and follow-up, or
3. achieving healthy dietary guidelines of DQT scores >60% [62] at baseline, post program, and follow-up.

### Secondary Outcome Measures

#### Smoking Rates

Among smokers, the proportion of people who have quit smoking will be compared for the 2 groups.

#### Change Over Time in Other Psychosocial and Attitudinal Measures

Change over time in scores on the GAD-7, WHO-5, and SF-12 and in self-efficacy and readiness to change behaviors and emotions will be compared between the self-directed and supported groups using repeated-measures analysis of variance. Multiple regression analysis, with groups entered as potential predictors, will be used to predict participants with greatest improvements in GAD-7, WHO-5, SF-12, and self-efficacy scores, and readiness to change behaviors and emotions.

### Assessing the Back on Track Program Acceptability

Proportions will be calculated for all items on the program acceptability scale.

## Results

Recruitment began in May 2020 and concluded in August 2021. A total of 122 participants were enrolled in this study. Data collection, including 2- and 6-month follow-up is expected to be completed by February 2022, and data analysis and publication of results will be completed by June 2022.

## Discussion

With cardiac event survivors at a heightened risk of a recurrent events and premature death, and with suboptimal attendance at center-based rehabilitation and secondary prevention programs, there is a need for innovative eHealth programs to support people in their behavioral and emotional self-management after an acute cardiac event. We anticipate that the *Back on Track* program will assist people in their recovery after an acute event and will represent a flexible, easily accessible, user-friendly, and effective adjunct to center-based programs. Our findings will enable us to quantify the behavioral and emotional improvements obtained and sustained for people with CHD who participate in the *Back on Track* program, while also comparing two methods of delivery: one fully self-directed and the other supported by a lifestyle counselor**.**

## Abbreviations

AAS: Active Australia Survey
ACHH: Australian Centre for Heart Health
AMI: acute myocardial infarction
ANCOVA: analysis of covariance
CABGS: coronary artery bypass graft surgery
CBT: cognitive-behavioral therapy
CHD: coronary heart disease
CR: cardiac rehabilitation
DQT: Diet Quality Tool
GAD-7: 7-item Generalized Anxiety Disorder instrument
IPAQ: International Physical Activity Questionnaire
ITT: intention to treat
MI: motivational interviewing
NHFA: National Heart Foundation of Australia
PCI: percutaneous coronary intervention
PHQ: Patient Health Questionnaire
PICF: participant information consent form
RCT: randomized controlled trial
REDCap: Research Electronic Data Capture
SF-12: Short Form Health Survey
WHO-5: World Health Organization–Five Well-Being Index
